# Continuous pressure measurement and serial micro–computed tomography analysis during injection laryngoplasty: A preliminary canine cadaveric study

**DOI:** 10.1371/journal.pone.0239544

**Published:** 2020-09-23

**Authors:** Min-Su Kim, Seongmin An, Songwan Jin, Taehoen Kim, Tack-Kyun Kwon

**Affiliations:** 1 Department of Otorhinolaryngology–Head and Neck Surgery, CHA Bundang Medical Center, CHA University School of Medicine, Seongnam, Gyeonggi-do, Republic of Korea; 2 Department of Mechanical Engineering, Korea Polytechnic University, Siheung, Gyeonggi-do, Republic of Korea; 3 Department of Pathology, CHA Bundang Medical Center, CHA University School of Medicine, Seongnam, Gyeonggi-do, Republic of Korea; 4 Department of Otorhinolaryngology–Head and Neck Surgery, Seoul National University College of Medicine, Seoul, Republic of Korea; University Hospital Eriangen at Friedrich-Alexander-University Erlangen-Numberg, GERMANY

## Abstract

Injection laryngoplasty (IL) has been used to treat various types of glottal insufficiency. The precise volume and location of the injected materials impact the outcomes. However, exactly how increasing volumes of material are distributed is unknown. In fact, the amount of IL material required to medialize a vocal cord tends to be determined empirically. Thus, the goal of this study was to investigate the pattern of IL material distribution by checking serial micro–computed tomography (MCT) and pressure changes during ILs. This experimental study used 10 excised canine larynges. Experimental devices included the IL syringe, pressure sensor, infusion pump, fixed frame, and monitoring system. We injected calcium hydroxyapatite in the thyroarytenoid muscle; whenever 0.1 mL of material was injected, we obtained an MCT scan while simultaneously measuring the pressure. After the experiments, we performed histologic analyses. MCT analyses showed that materials initially expanded centrifugally and then expanded in all directions within the muscle. The pressure initially increased rapidly but then remained relatively constant until the point at which the materials expanded in multiple directions. Histologic analyses showed that the IL material tended to expand within the epimysium of the thyroarytenoid muscle. However, in some cases, the MCT revealed that there were leakages to the surrounding space with a corresponding pressure drop. If the IL material passes through the epimysium, leakage can occur in the surrounding space, which can account for the reduction in resistance during ILs.

## Introduction

Injection laryngoplasty (IL) has been used worldwide to treat various types of glottal insufficiency. It is known to be effective for preventing aspiration pneumonia and improving the voice in glottal insufficiency, such as in unilateral vocal paralysis [1]. Many types of injection materials have been developed and selected for different purposes, with accompanying reports on these materials’ effectiveness and safety. Although IL is now widely performed with many materials, the techniques used are highly variable [2–4]. Outcomes after ILs rely on the precise volume and location of the injected material; the volume and location can be challenging to control, especially for beginners, because of the pressure required to inject a viscous substance through a small needle.

In fact, there are some unresolved questions for laryngologists. First, information on how the material is distributed within the larynx during an IL is limited. This information is needed to make decisions about where to place the injection syringe needle and how much material should be injected during an IL. Second, when the injection volume is low, many laryngologists find that the material disappears after a short time. However, to our knowledge, the redistribution pattern of the injected material has rarely been investigated scientifically. Third, the force required to push an injection syringe plunger initially increases as the amount of material injected increases; then, at some point, it is possible to inject the material with less force.

This study was developed to solve these questions that have arisen from clinical experience during ILs. This study is a preliminary report on experimental tools that simultaneously measure both the distribution of the injected material and the change in injection pressure, while assuming the injection force needed to push the plunger of the injection syringe during an IL.

## Materials and methods

This study was approved by the Institutional Animal Care and Use Committee of the Seoul National University Hospital (number 16-0208-S1A0) and was conducted in strict compliance with its requirements and determinations. A total of 16 larynges were excised from 16 Beagle dogs. The larynges were extracted from the carcasses after the completion of another canine experiment for a wearable cardiac pacemaker, which was also approved by the Institutional Animal Care and Use Committee (number 15-0313-S1A0) and was conducted through a specialized experimental animal company (Orient Bio, Seongnam, Republic of Korea). Of the 16 canine larynges, 6 were used in the pilot study for the development of the infusion pump and fixation device used in the micro–computed tomography (MCT). The other 10 canine larynges were used in the main experiment.

### Preparation of experimental devices

**Fig 1:**
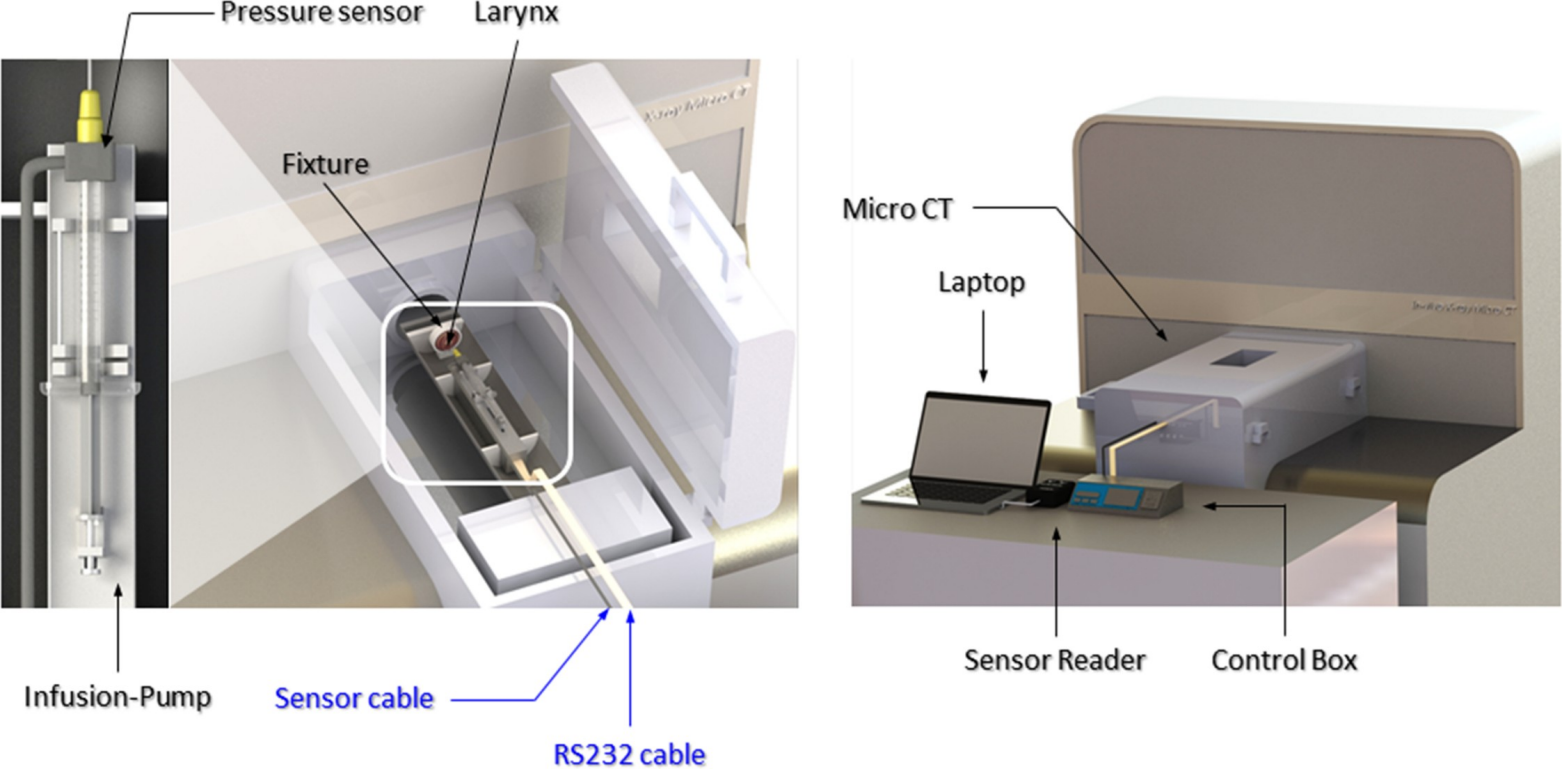
Schematic figure showing the experimental setup.

ILs were performed in the excised canine larynges using an infusion pump (NanoJet, Chemyx, Stafford, TX, USA) at a constant injection flow rate. The infusion pump comprised an infusion pump header and a control box. The infusion pump header was responsible for pushing the plunger of the injection syringe and was located inside the MCT in order to continuously measure pressure during the IL. It was connected to the control box through wires outside of the MCT. The control box set up the flow rate and monitored the status of the injection. The pressure sensor (ELVEFLOW, Paris, France) was located between the needle and the syringe, and was connected to the laptop through wires (RS232 cables). Continuous pressure was measured during the experiments. Analog pressure data were converted to digital data using a sensor reader between the pressure sensor and the laptop. We analyzed the collected pressure data during the experiments in real time using ELVEFLOW software. The pressure data shown in the results are the absolute pressure minus the atmospheric pressure. To prevent them from shaking, we used a device to fix the canine larynx and infusion pump header in the MCT tray (Fig 1).

This device was constructed using three-dimensional (3D) printing and designed with Solidworks software (Dassault Systems, Vélizy-Villacoublay, France). The designed models were converted to stereolithography files and imported into Cubicreator 3D printing system software for prototyping (Cubicon Single Plus, Seongnam, Republic of Korea). All of this work was conducted through collaboration with 2 authors who are mechanical engineers (SA and SJ).

### Experimental protocol

This experiment design is comparable to a transoral-style IL. The infusion pump was connected to the syringe for the IL (Fig 1). We used calcium hydroxyapatite (CAHA; Radiesse Voice, Merz Aesthetics, Frankfurt, Germany) as the injection material in the experiments, which were all performed by the same author (M-SK). Using a 25-gauge, 1.5-inch needle, the author injected the CAHA into the thyroarytenoid (TA) muscles of the excised canine larynges, which were in the fixation device. The author injected the CAHA into the middle portion of each TA muscle. The needle was inserted into the lateral aspect of the vocal ligament. The CAHA was injected continuously into the TA muscle under a constant flow rate by the syringe connected to the infusion pump. The MCT visualization experiment was performed while measuring the pressure. MCT scans were taken whenever 0.1 mL of CAHA was injected, and the pressure was measured simultaneously in real time.

### MCT and histological analysis

An MCT scanner (NFR Polaris-G90; NanoFocusRay, Jeonju, Republic of Korea) was used to identify the injected material in the cadaveric canine larynx model. There were 600 MCT images, which were taken at settings of 65 kv, 60 μA, and 500 ms with a resolution of 512 × 512 in Digital Imaging and Communication in Medicine format. We serially analyzed the diffusion pattern of the injected material. Specifically, the MCT recorded the anatomic site and surrounding area whenever 0.1 mL of material was injected. After each experiment, the canine larynges were fixed in formaldehyde, decalcified with ethylenediaminetetraacetic acid, cut into 0.5-cm slices, and embedded in paraffin blocks. Sections were stained for the histological analysis using hematoxylin and eosin.

## Results

**Fig 2:**
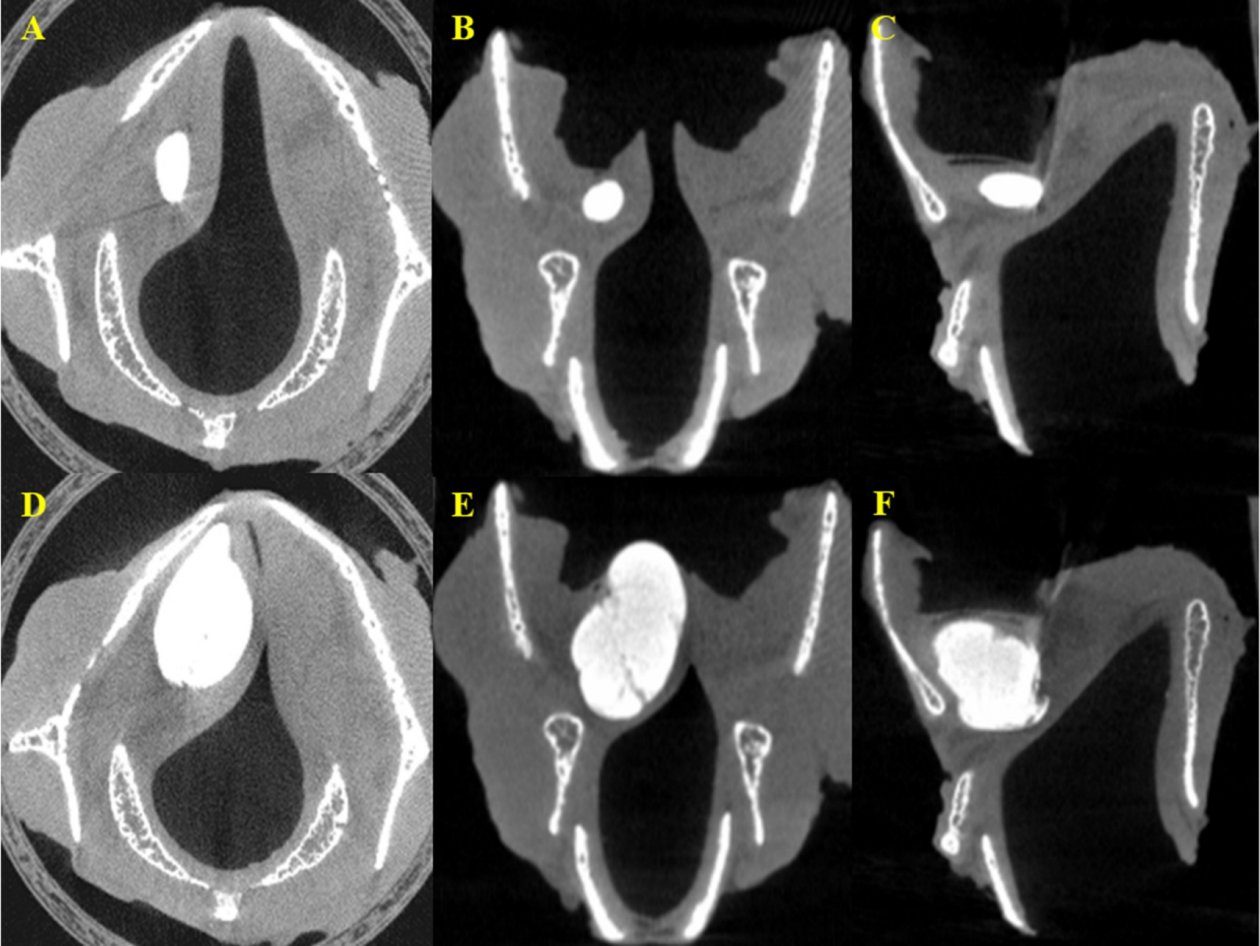
Serial view of micro–computed tomography. (A) Axial view at initial injection. (B) Coronal view at initial injection. (C) Sagittal view at initial injection. (D) Axial view before leakage. (E) Coronal view before leakage. (F) Sagittal view site before leakage.

The interval between 0.1-mL injections ranged from 67 to 123 seconds (median, 95 seconds). The time taken for injection ranged from 7.0 to 7.5 seconds (median, 7.2 seconds). The MCT analysis showed that in the early stages (0.1–0.2 mL) of IL, the CAHA proceeded in a centrifugal and spherical expansion around the needle tip (Fig 2A–2C). As the IL continued, the spherical expansion resulted in a medial bulging of the vocal fold but was blocked by the vocal ligament and thyroid cartilage. As the IL proceeded, the CAHA began to expand within the TA muscle, moving forward, backward, up, and down from the injection point. The downward expansion stopped at the cricothyroid space, and the superior expansion caused bulging to the ventricle (Fig 2D–2F).

**Fig 3:**
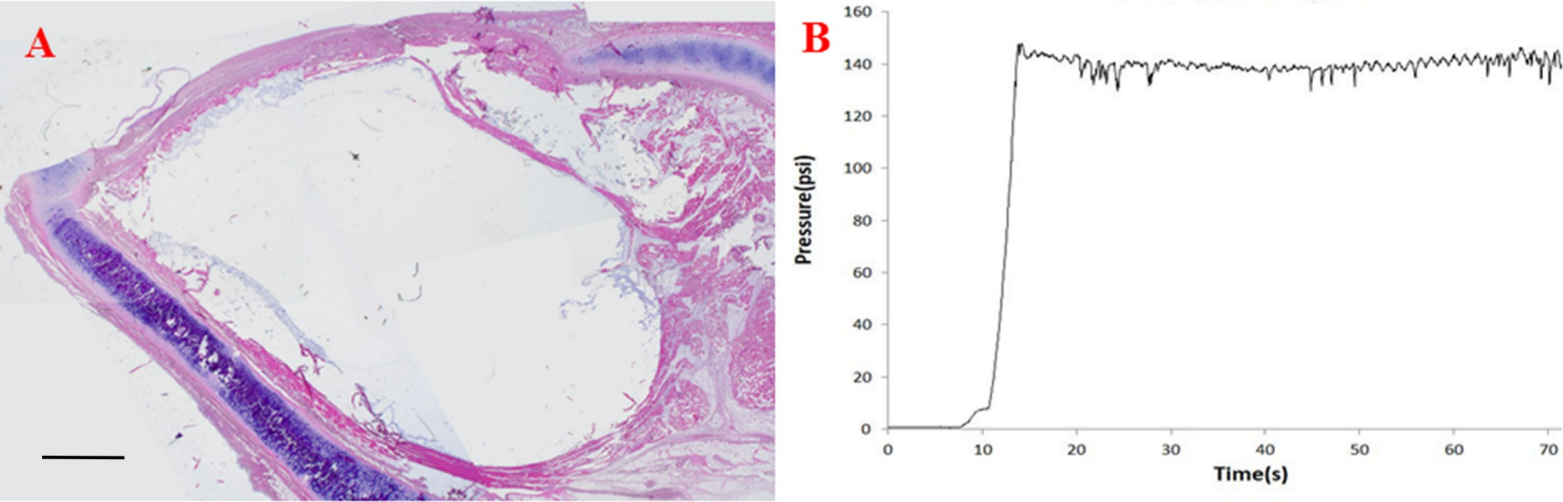
Analysis of the situation before leakage to the surrounding space. (A) Histologic analysis. Bar: 1 mm. (B) Pressure measurement.

Histologic examinations showed that the CAHA was dilated within the TA muscles. It expanded in an oval pattern within the muscle because it was blocked by the thyroid cartilage and the vocal ligament (Fig 3A). The pressure initially increased rapidly but then remained relatively constant (Fig 3B). If the CAHA did not leak, 1.3 mL was injected.

**Fig 4:**
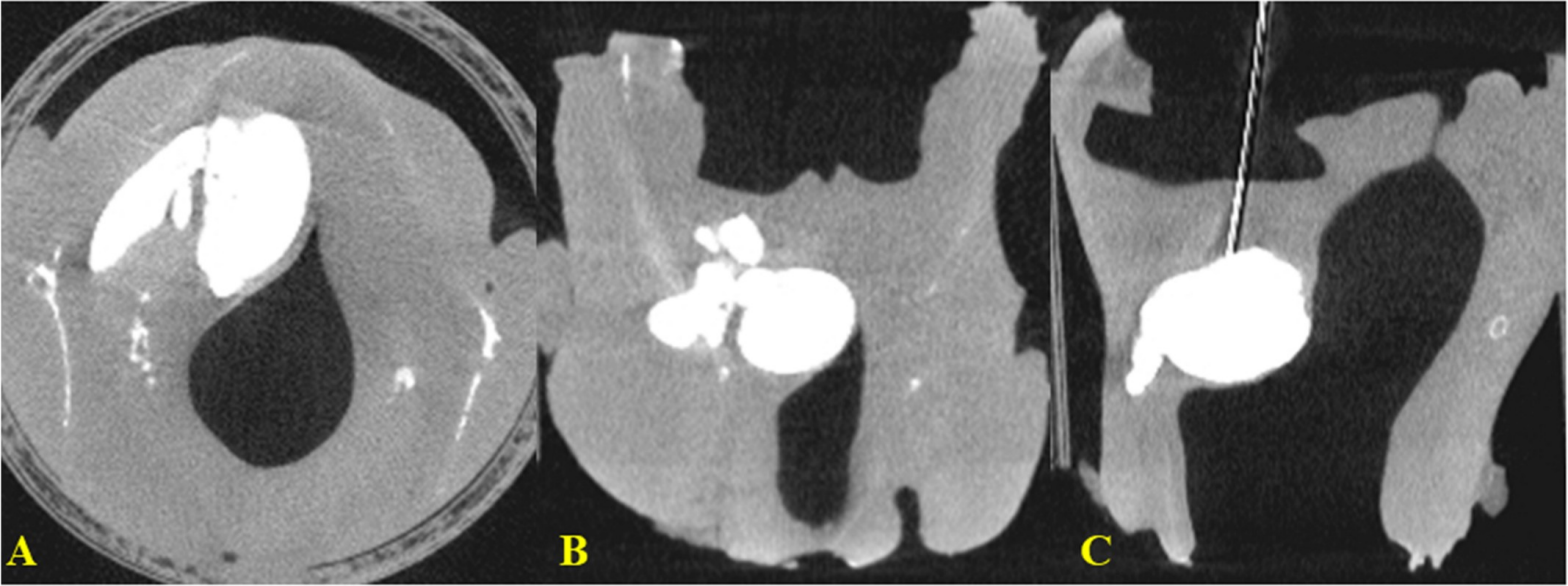
Serial view of micro–computed tomography after paraglottic leak occurred. (A) Axial view. (B) Coronal view. (C) Sagittal view.

All 10 injection experiments showed the same pattern as described above before leaks occurred in some cases. However, CAHA leaked to the surrounding spaces in some cases during the IL. In 1 case, there was a leak to the paraglottic space (Fig 4). In other cases, we observed leaks to the airway through the hole in which the injection needle was inserted.

**Fig 5:**
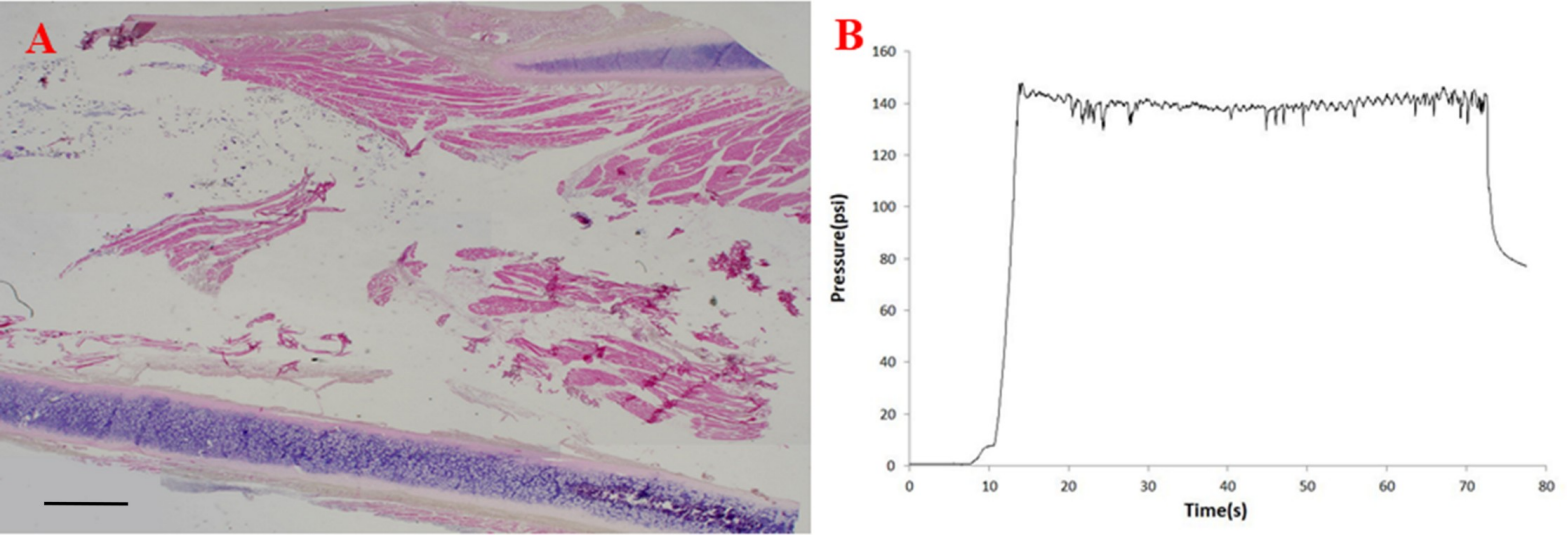
Analysis of the situation after leakage to the surrounding space. (A) Histologic analysis. Bar: 1 mm. (B) Pressure measurement.

In those cases, histologic examinations showed that the CAHA leaked when there was a TA muscle rupture (Fig 5A). The pressure initially increased rapidly, remained relatively constant, and then dropped rapidly after leakage (Fig 5B). When pressure dropped due to a leak, 0.9 to 1.1 mL of CAHA had been injected.

## Discussion

In a previous study, the safety of IL with CAHA was reported in nearly 1,000 cases [5]. Among the various materials used for IL, CAHA is widely used for long-term vocal fold augmentation. CAHA is easily visualized in computed tomography (CT) because the calcified material is radiopaque. We previously reported that CAHA can be adequately observed in CT as a bright whitish mass in the glottic and paraglottic space [6]. Therefore, CAHA is the best material to use when performing a visual analysis of the distribution pattern of injected material. However, it is ethically problematic to conduct serial CT while simultaneously performing an IL in the human body. Therefore, we performed this study using excised canine larynges instead. The canine larynx is widely used as an appropriate model for studying human laryngeal physiology, because the size and histological properties of the canine and human larynxes are similar [3, 7–9]. In addition, in our experiment the volume and velocity of the injection were controlled by a modified syringe pump. The force required to push a plunger can vary based on the size of the syringe and needle. The force is calculated by multiplying the pressure by the area. The area of the syringe containing the injection material is constant. Therefore, we measured the pressure changes in the syringe, which represent the changes of force. The most problematic part of this study was the simultaneous installation of the excised canine larynx, fixation device, syringe, pressure sensor, and syringe pump inside the limited space of the MCT tray in order to record data from the devices in real time. A transcutaneous-style IL experiment could not be simulated because of the short height of the MCT tray space, as the syringe pump with the IL syringe had to be mounted diagonally. Thus, because of the narrow MCT tray space, the simulation used a transoral-style IL experiment, in which the needle passes horizontally through the upper part of the vocal cords.

From the results of this experiment, we can interpret information about the distribution of injection material. First, in the early stages of an IL, the injection material expanded spherically in a centrifugal direction because the IL material initially pushed outward within the TA muscle. However, as the amount of IL material increased, it started to expand in an oval pattern along the longitudinal axis of the vocal cord, where the resistance is low; this is probably because the vocal ligament, conus elasticus, and thyroid cartilage acted as barriers. A histologic analysis showed that the injection material stayed in the TA muscle space in most cases. However, when the IL material exceeded a certain amount, it leaked to the paraglottic space from the TA muscle. We assume that the intramuscular pressure exceeded the elastic limit of the epimysium of the TA muscle at this point, which resulted in a rupture at its weakest area that, in turn, allowed the material to leak into the surrounding space. When the leakage to the surrounding space occurred, an additional injection might not affect the medialization of the vocal cord. Our histological analysis showed how CAHA leaked out of the TA muscle and into the paraglottic space. We assume that once CAHA leaks to the paraglottic space, it can be redistributed to the base of the pyriform sinus, aryepiglottic fold, or postcricoid space, which are connected posteriorly by loose areolar tissue. This assumption explains why we sometimes find a lump of injection material in the posterior cricoid space during the arytenoid adduction procedure after a CAHA injection. Some studies have shown similar results. Mau et al performed a transoral-style IL experiment with CAHA in human cadaveric larynges, checking the CT scans both before and after the IL. The results showed that the CAHA tended to have a relatively compact appearance on CT and was distributed along the length of the vocal fold when injected medially. By contrast, in 20% of female cadaver larynges (3/15 cases), CAHA leaked through the cricothyroid space outside of the laryngeal framework when injected laterally [10]. However, our results did not show leakage to the cricothyroid space. The difference in these results is believed to be due to the use of the relatively larger canine larynx with a lateral injection, as compared to a human female larynx without a lateral injection. In addition, the loss of the medialization effect after a short time can be attributed to the redistribution of injection material to the surrounding spaces during the IL.

Second, if the above hypothesis is correct, it is possible that the abrupt reduction of resistance to the syringe during the IL procedure is a result of an epimysium rupture. The resistance felt by a laryngologist during an IL may have various causes. The most common cause of resistance is clogging of soft tissue or cartilage in the needle when the operator begins to push the plunger. However, the resistance decreases once the IL proceeds. Resistance caused by needles approaching the arytenoid cartilage or the vocal ligament in close proximity is also common. In this situation, the resistance can decrease if the needle is repositioned. However, in the present study we experienced a decrease in resistance during injection, unlike in the scenarios described above. Once the resistance decreases, the remaining material is injected very easily. This phenomenon may be represented as a pressure drop due to an epimysium rupture. In our experiment, some cases showed a sudden drop in pressure, which might support this hypothesis. Thus, our findings suggest that the IL material escapes somewhere, moving away from the TA muscle.

This study has some strengths. First, this was a nearly real-time analysis of IL, which has rarely been conducted. Second, we accurately and scientifically measured injection pressures. Third, we analyzed MCT serially and variously (i.e., in the axial, coronal, and sagittal views). This visual analysis enabled a profound understanding of the distribution patterns of the injection material.

The present study also has some limitations. First, we did not simulate a transcutaneous-style experiment. Future transcutaneous-style experiments may demonstrate a pattern of injection material distribution that differs from the current results. Second, because of the time needed for MCT scanning, a step-by-step injection method was used, which can result in a different outcome than an actual clinical continuous injection. Third, we did not investigate various IL materials, such as hyaluronic acid or carboxymethylcellulose. Each material has unique viscosity and resorption characteristics [11]. Therefore, results with different IL materials may differ from the findings of this study. Finally, the canine larynx is different from the human larynx, although it is considered to be a good experimental alternative to the human larynx. In addition, we used frozen and thawed canine larynges, which may react differently from non-frozen canine larynges. In the future, we will conduct a more controlled and improved experiment. It is possible to verify our hypothesis by increasing the number of experimental objects and creating a transcutaneous-style model to observe the distributed patterns.

## Conclusions

The IL material expands within the epimysium of the TA muscle, and its range is limited in the vocal ligament, conus elasticus, and thyroid cartilage. However, if the injection material passes through the epimysium, leakage can occur in the surrounding space, which can account for the reduction in resistance during an IL.
